# MIS-C Treatment: Is IVIG Always Necessary?

**DOI:** 10.3389/fped.2021.753123

**Published:** 2021-11-03

**Authors:** Francesco Licciardi, Letizia Baldini, Marta Dellepiane, Carlotta Covizzi, Roberta Mogni, Giulia Pruccoli, Cecilia Orsi, Ivana Rabbone, Emilia Parodi, Federica Mignone, Davide Montin

**Affiliations:** ^1^Department of Pediatrics and Public Health, Università degli Studi di Torino, Turin, Italy; ^2^Ospedale Infantile Regina Margherita, Città della Salute e della Scienza, Turin, Italy; ^3^Postgraduate School of Pediatrics, Università degli Studi di Torino, Turin, Italy; ^4^Department of Health Sciences, University of Piemonte Orientale, Novara, Italy

**Keywords:** MIS-C, SARS-CoV-2, therapy, IVIG (intravenous immunoglobulin) administration, steroid

## Abstract

**Background:** MIS-C is a potentially severe inflammatory syndrome associated with SARS-CoV-2 exposure. Intravenous immunoglobulin (IVIG) is considered the first-tier therapy, but it implies infusion of large fluid volumes that may worsen cardiac function.

**Patients and Methods:** Since April 2020, we have developed a treatment protocol that avoids the infusion of IVIG as first-line therapy in the early phase of MIS-C. In this study, we retrospectively analyzed a cohort of consecutive patients treated according to this protocol between 01/04/2020 and 01/04/2021.

**Results:** In the last year, 31 patients have been treated according to the protocol: 25 with high-dose pulse MP (10 mg/kg) and 6 with 2 mg/kg. 67.7% of the patients responded to the initial treatment, while the others needed a step-up, either with Anakinra (25.8%) or with MP dose increase (6.5%). IVIG was administered in four patients. Overall, only one patient (3.2%) needed ICU admission and inotropic support; one patient developed a small coronary artery aneurysm.

**Conclusions:** Timely start of MP therapy and careful fluid management might improve the outcomes of MIS-C patients.

## Introduction

Multisystem inflammatory syndrome in children (MIS-C) is a newly described disease, characterized by fever, abdominal pain, lymphopenia, myocardial dysfunction, and some additional clinical features—such as conjunctival injection, oral erythema, and cutaneous rash—which are also typical of Kawasaki Disease (KD) (1).

Since the very first reports, MIS-C has been temporally linked to SARS-CoV-2 exposure. At present, even if the pathogenesis remains unknown, an increasing body of epidemiological and biochemical evidence supports the hypothesis that it is a post-infectious process developing 4–6 weeks after SARS-CoV-2 infection (2).

MIS-C can be a severe disease, requiring ICU admission in up to 73.3% of the patients and causing the development of coronary artery anomalies (CAA) in up to 21.9% of the patients (3). The partial clinical overlapping has led most physicians to treating MIS-C like KD; hence, intravenous immunoglobulin (IVIG) is the most frequently prescribed first-tier therapy in MIS-C (1). Nevertheless, IVIG administration implies infusion of large fluid volumes (40 ml/kg) that may worsen the myocardial dysfunction in the early phase of disease (4).

Since April 2020, at OIRM (Ospedale Infantile Regina Margherita, Turin, Italy), we have been treating MIS-C patients with intravenous methylprednisolone (MP) alone, adding subcutaneous Anakinra as a step-up therapy, in order to decrease the need of IVIG during the first days after disease onset. The aim of the study is to analyze the outcomes of the patients treated according to our treatment protocol, between April 2020 and April 2021.

## Methods

### Setting and Treatment Protocol

OIRM is the main pediatric hospital in Piedmont, a region in Northwest of Italy (4,341,375 inhabitants, 723,208 <20 years of age) (5). At OIRM, all the patients suspected to have MIS-C undergo a comprehensive diagnostic work-up, including cell blood count, C-reactive protein (CRP), serum IgG, and RT-PCR on nasal swab for SARS-CoV-2, NT-pro-B-type natriuretic peptide (NT-pro-BNP), and echocardiogram. Patients satisfying the preliminary case definition of MIS-C (6) and having positive SARS-CoV-2 IgG are treated according to an internal step-up treatment protocol that does not include IVIG as first-tier therapy.

**Figure 1:**
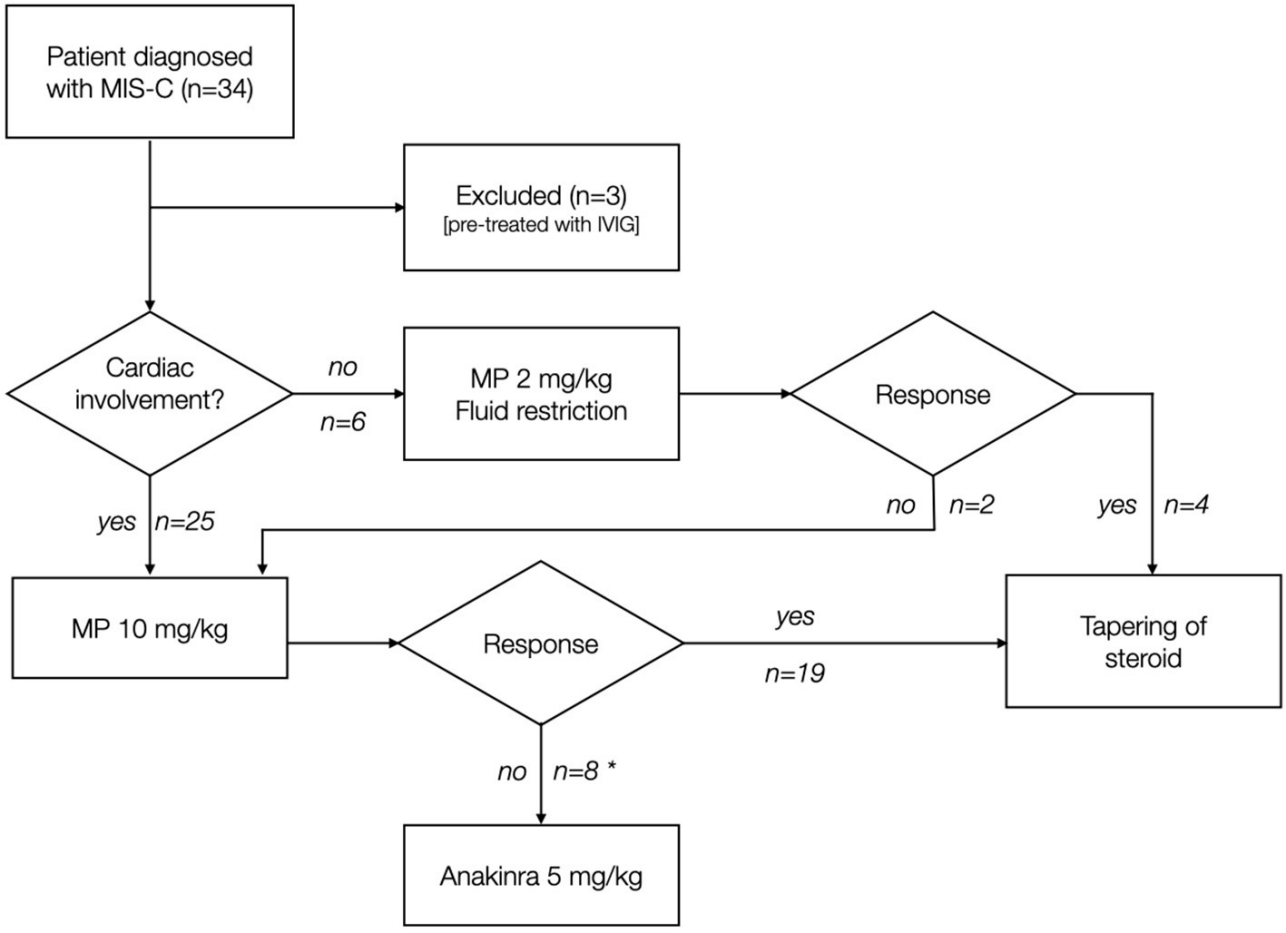
Treatment strategy of patients included in the study. MP was used in patients as first-tier monotherapy. Clinical response was defined as defervescence and CRP reduction after 48 h from the first MP dose. *4 patients received IVIG.

The initial treatment in all the patients is MP: if myocardial involvement is present [hypotension according to age-, gender-, and height-adjusted chart (7); or Ejection Fraction (EF) <50%; or NT-pro-BNP ≥1,500 pg/ml], the patient receives high-dose pulse IV MP 10 mg/kg/day (single dose) for 3–5 days; otherwise, low-dose IV MP 2 mg/kg/day (single dose) is administered. After 48 h, if CRP increases and/or fever persists, the treatment is intensified either with a MP dose increase or by adding subcutaneous Anakinra 5 mg/kg/day (Figure 1). After achieving clinical response, MP therapy is switched to oral prednisone before discharge and tapered over 4 weeks; Anakinra is tapered over 2 weeks. IVIG is reserved for patients with suspected CAA at any ultrasound evaluation [defined according to American Heart Association 2017 Guidelines for KD (8)], or presenting persistent symptoms despite defervescence and CRP reduction, or as a third-line therapy in patients unresponsive to MP and Anakinra. IVIG is infused over 18 h; in case of urine output decrease or worsening of cardiac function, the infusion speed is lowered. In case of concomitant severe heart failure, a 2-day IVIG infusion regimen is preferred (4).

For coronary monitoring, echocardiography is carried out by an experienced pediatric cardiologist every other day in the first week after admission, before discharge, and 6 weeks later.

Considering the high prevalence of myocardial dysfunction, all the patients with MIS-C undergo careful monitoring of fluid balance. We use the Holliday and Segar formula for calculating the minimal maintenance water needs, and oral hydration is preferred over intravenous liquid infusion, whenever possible. During the hospitalization, we tailor liquid intake considering multiple variables, such as daily weight variation, intake/output balance, and blood creatinine.

Regarding prophylactic anticoagulation, every patient diagnosed with MIS-C is evaluated according to an institutional risk assessment model: the patients with high risk receive enoxaparin 100 U/kg/day until discharge or thrombotic risk reduction, whichever comes first (9). Finally, anti-aggregation with acetylsalicylic acid (3–5 mg/kg/day) is prescribed when a CAA is documented or suspected during any US examination. The patients with persistent CAA receive anti-aggregation therapy according to American Heart Association 2017 Guidelines for KD (8).

### Study Design, Data Collection, and Statistical Analysis

We performed a retrospective analysis of the patients with MIS-C, treated according to the abovementioned protocol, between 01/04/2020 and 01/04/2021, at OIRM. The patients who had received IVIG prior to the admission to our hospital and those with negative SARS-CoV-2 serology were excluded from further analysis.

We divided the patients into two groups, according to the MP starting dose: high-dose pulse (Group A) or low dose (Group B). As primary outcomes, we considered the following: the rate of ICU admission after starting treatment, the rate of inotropic support needs after starting treatment, and the incidence of CAA. As secondary outcomes, we evaluated the following: the number of days between MP first dose and CRP halving, the number of days between MP first dose and NT-pro-BNP halving, and the days between first pathological echocardiogram and EF normalization.

The local ethical committee approved the data collection on March 24, 2020; an informed consent was obtained, in accordance with the Declaration of Helsinki.

Statistical analysis was performed by using GraphPad Prism 6.0. The differences between groups were analyzed using Mann–Whitney *U*-test for continuous variables and Fisher's exact test for categorical variables. All the tests were two tailed and the significance was set at *p* ≤ 0.05.

## Results

### Patients and Baseline Features

In the period considered, 34 patients diagnosed with MIS-C were treated at OIRM. Among these, three patients received IVIG as first-line therapy, so they were excluded from further analysis. Overall, 31 patients (91.2%) were treated according to the protocol; epidemiological, clinical, laboratory, and echocardiographic features at first evaluation are summarized in Tables 1, 2. The median time between disease onset and the first dose of MP was 5 (4–6) days. Twenty-five patients (80.7%) were treated with high-dose pulse MP (Group A): 32% had EF <50%, 100% had NT-pro-BNP >1,500 ng/ml, and 64% had hypotension.

**Table 1:** Demographic and clinical features of Group A and Group B.

	**Group A**	**Group B**	***p*-value**
Patients	25	6	–
Age (years)	8 (7–11)	9 (6–10.5)	0.90
Female	44.0%	50.0%	0.79
Ethnic group	84.0% White European 12.0% West African 4.0% South American	83.3% White European 16.7% South American	1.0
Previous positivity of SARS-CoV-2 swab	36.0%	33.3%	0.90
Close relative positive for SARS-CoV-2	76.0%	83.3%	0.70
Fever >38°C	100%	100%	1.00
Days of fever	5 (4–6)	4 (3–6)	0.42
Conjunctivitis	96.0%	66.7%	0.09
Mucositis	44.0%	66.7%	0.39
Lymphadenitis	48.0%	33.3%	0.66
Hand or feet lesions	36.0%	66.7%	0.21
Rash	72.0%	50.0%	0.36
Abdominal pain	76.0%	66.7%	0.63
Diarrhea	24.0%	33.3%	0.63
Vomit	60.0%	16.7%	0.08
Hypotension^§^	64.0%	0%	**0.007**

§ *According to age-, gender-, and height-adjusted chart (7)*.

*Continuous variables are described as median and interquartile range*.

*Bold font indicates statistical significance*.

**Table 2:** Laboratory features of Group A and Group B.

	**Group A**	**Group B**	***p*-value**
WBC count (/mm^3^)	9,590 (6,490–12,160)	6,590 (6,338–7,195)	0.19
Lymphocyte (/mm^3^)	780 (450–1,080)	695 (618–773)	0.83
Neutrophil (/mm^3^)	8,410 (5,080–10,370)	5,815 (5,190–8,460)	0.44
Platelet (*1,000/mm^3^)	131.0 (106.0–180.0)	132.5 (99.3–165.0)	0.78
CRP (mg/L)	224 (125–316)	179 (150–209)	0.89
PCT (ng/ml)	10.3 (3.7–33.1)	12.7 (4.6–20.5)	0.37
Ferritin (ng/ml)	634 (493–1078)	342 (278–461)	0.08
ESR (mm/h)	49 (32–54)	58 (51–61)	0.10
Albumin (g/dl)	3.2 (2.8–3.4)	3.4 (3.1–3.9)	0.61
ALT (UI/L)	44 (19–58)	24 (18–32)	0.14
AST (UI/L)	36 (27–57)	29 (27–41)	0.38
Na (mmol/L)	131 (129–132)	133 (132–136)	0.08
Creatinine (mg/dl)	0.47 (0.41–0.62)	0.53 (0.33–0.65)	0.99
INR	1.22 (1.07–1.35)	1.34 (1.22–1.45)	0.60
aPTT ratio	0.96 (0.91–1.1)	1.01 (0.93–1.03)	0.80
Fibrinogen (mg/dl)	631 (573–700)	628 (493–650)	0.56
D-dimer (ng/ml)	4,284 (2,548–4,906)	2,058 (1,548–3,210)	0.13
Positive swab for SARS-CoV-2	12.0%	16.7%	1.00
IgG against SARS-CoV-2	100%	100%	1.00
Ejection fraction <50%	32.0%	0%	0.29
NT-proBNP pg/ml	8,231 (4,586–12,571)	698 (147–1,197)	** <0.001**
NT-proBNP >1,500 pg/ml	100.0%	0%	** <0.001**
Troponine (ng/L)	48 (27–120)	9 (8–29)	0.09

*Continuous variables are described as median and interquartile range*.

*Bold font indicates statistical significance*.

Patients of Group A and Group B had similar features at disease onset (Tables 1, 2), except for the baseline pro-BNP level (8,231 vs. 698 pg/ml, *p* < 0.001) and the prevalence of hypotension (64 vs. 0%, *p* = 0.007). Fifty-two percent of Group A patients received high-dose pulse IV MP 10 mg/kg/day for 5 days, while the others were treated for 3 days.

### Post-treatment Course and Outcomes

Eight patients of Group A (32%) needed a step-up by Anakinra 5 mg/kg/day due to persistent fever (87.5%) or CRP increase (12.5%); four patients (12.9%) received IVIG: one due to persistent irritability despite satisfactory CRP decrease and fever resolution, two due to a suspicion of CA dilatation, not confirmed by the follow-up ultrasound (*z*-score always <1.5), and one due to the development of a small right CA aneurysm (5 mm, *z*-score 4). The mean time between the first MP dose and IVIG administration was 5 days.

Six patients (19.3%) were treated with low-dose MP (Group B), and two (33.3%) of them needed MP dose increase due to persistence of fever (Figure 1).

After clinical response was achieved, MP therapy was switched to oral prednisone, and tapered over 4 weeks in all the patients. The median follow-up after disease onset was 133 days (IQR 95–163).

All the patients recovered: detailed outcomes are described in Table 3. Overall, one patient (3.2%) needed ICU admission and inotropic support, and one developed a CAA 6 days after the start of MP. Median CRP halving time was 2 days (2 days in Group A and 5 days in Group B), NT-pro-BNP halved in 3 days in Group A, while EF normalized in 5 days. Acute kidney injury was observed in one patient of Group A.

**Table 3:** Outcomes and concomitant hematological treatment of Group A, Group B, and overall cohort.

	**Group A**	**Group B**	**Total**
Patients	25	6	31
MP response^*^	68.0%	100%^**^	74.2%
ICU admission	4.0%	0%	3.2%
Inotrope support	4.0%	0%	3.2%
Acute kidney injury^#^	4.0%	0%	3.2%
Coronary artery anomalies	4.0%	0%	3.2%
CRP halving time	2 (2–3) days	5 (3–5) days	2 (2–3) days
NT-proBNP halving time	3 (2–5) days	–	3 (2–5) days
EF normalization time	5 (4–5) days	–	5 (4–5) days
**Concomitant hematological treatment**			
LMWH^§^	52.0%	0%	41.9%
Acetylsalicylic acid^°^	12.0%	0%	9.7%

*Continuous variables are described as median and interquartile range*.

* *Defined as defervescence and CRP reduction after 48 h from the first MP dose*.

** *Two patients needed dose adjustment*.

# *According to Kidney Disease Improving Global Outcomes definition for AKI (10)*.

° *Acetylsalicylic acid was prescribed when a suspicion of coronary abnormality was suspected during US examination*.

§ *Low-molecular-weight heparin was prescribed according to an institutional risk assessment model (9)*.

## Discussion

MIS-C is an emerging inflammatory syndrome affecting children a few weeks after SARS-CoV-2 exposure, which shares some features with KD. No treatment trial in MIS-C has been published so far, but IVIG is the most widely adopted treatment (1). IVIG therapy was borrowed from KD; nevertheless, its use in the early phase of MIS-C raises some concerns. Firstly, IVIG administration implies infusion of large volumes (40 ml/kg) that may cause heart overload especially in patients with myocardial dysfunction (4). Moreover, IVIG increases oncotic pressure and consequently causes liquid shift, which may further worsen fluid overload in these patients, who often present hypoalbuminemia and third-spacing. As far as we know, this is the first report of MIS-C patients treated according to an IVIG-sparing protocol, developed to avoid IVIG infusion in the early phase of the disease, when the risk of heart failure is high.

The outcomes of the patients treated according to the above-described protocol (see Method) are rather encouraging. Notably, only one patient among 31 needed ICU admission and inotropic support. Regarding CAA, only one patient (3.2%) developed a small aneurysm (*z*-score = 4), which is stable 4 months after discharge. Interestingly, the only patient who developed CAA was treated later than the others (8 vs. 5 days), suggesting that a timely start of MP may be protective. The positive outcomes of our cohort could not be attributed to a selection bias. Indeed, the baseline features of our cohort are comparable to other MIS-C cohorts; notably, the rate of patients with myocardial involvement is high (81%), with a mean pro-BNP level at disease of 6,540 pg/ml (11). Several factors might explain our results. First, the vast majority of our patients received MP in the earliest phase of the disease. A recent multi-omics study in MIS-C suggests that IV glucocorticoids have a rapid effect on decreasing the level of many soluble biomarkers associated with type II IFN response (IFN-γ, CXCL9), T-cell activation (sCD25), cell adhesion (sE-Selectin/sCD62E), and monocyte/macrophage activation (sTNFRII, M-CSF, and ferritin, IL-6); this evidence may support the utility of steroids in MIS-C treatment (12).

So far, three real-life retrospective studies have been conducted regarding the use of steroids to treat MIS-C (2, 11, 13). In two of them, the authors compared the outcomes of the patients treated with steroids and IVIG vs. IVIG alone. Ouldali et al. found that adding MP to IVIG led to a significant decrease of hemodynamic support needs and a reduction in length of ICU stay; Son et al. found that the patients treated with steroids and IVIG had a lower risk of new or persistent cardiovascular dysfunction compared to the ones treated with IVIG alone (2, 11).

The effect of steroid treatment without IVIG has been less studied. As far as we know, only a recent paper by the BATS consortium describes the outcomes of a cohort treated with steroids as a first-line monotherapy. The authors report that the inotropic or ventilator support rate of the cohort was 17.2%. Unfortunately, the data collected by the BATS consortium were obtained in patients treated with non-homogeneous protocols; therefore, a direct comparison with our results is not possible (13). Of note, in the BATS study, 47.5% of the patients treated with steroids also received IVIG in the days following disease onset, and none received Anakinra. The explanation for the differences of the BATS outcomes and ours may lay in the second-line therapies: Anakinra in our protocol, IVIG in BATS (13). Furthermore, our fluid management (see Materials), aimed at decreasing the risk of overload, may have further reduced the ICU admission rate.

Our study has some limitations, including the sample size and the absence of a control group, which hampers firm conclusions regarding the comparison with IVIG-based protocols. Moreover, the comparison with previously published cohorts may be affected by different inclusion criteria, as we only enrolled patients with SARS-CoV-2-positive serology. Nevertheless, the very first published MIS-C cohorts showed much higher rates of ICU admission (73.3%), inotropic support (55.3%), and aneurysm incidence (10.3%) (3). These early reports might have been affected by the poor knowledge of MIS-C in 2020, and current outcomes might have improved worldwide.

Despite its limitations, our study highlights the importance of a tailored step-up treatment of MIS-C. As a first-line therapy, MP alone seems a good option, especially in countries where IVIG is not available. The response rate to MP in our cohort was satisfactory, but 25.8% of the patients needed further step up. To treat patients with persistence of fever and/or CRP increase, we preferred Anakinra to IVIG, due to its low effect on fluid overload. All the patients treated with IL-1 receptor antagonist had a clinical and laboratory response within 48 h. Even if IVIG protective role against CAA formation has never been proven in MIS-C, infusing IVIG 2 g/kg seems a reasonable option in patients showing CAA at any time point (1). In case of IVIG infusion, great attention should be paid to fluid overload, especially in the earliest days of the disease (1, 4).

In conclusion, our results suggest that favorable outcomes in MIS-C could be achieved by sparing IVIG infusion in the earliest phase of the disease; compared to previously published studies, the incidence of CAA does not increase. A prospective randomized controlled trial should be designed, to define the effective role of IVIG and steroids in MIS-C treatment.

## Data Availability Statement

The raw data supporting the conclusions of this article will be made available by the authors, without undue reservation.

## Ethics Statement

The studies involving human participants were reviewed and approved by Comitato Etico Interaziendale A.O.U. Città della Salute e della Scienza di Torino—A.O. Ordine Mauriziano—A.S.L. Città di Torino. Written informed consent to participate in this study was provided by the participants' legal guardian/next of kin.

## Author Contributions

FL conceived the study, wrote the statistical analysis plan, analyzed the data, and drafted and revised the paper. LB monitored data collection, designed graphic figures, and drafted and reviewed the paper. MD, CC, RM, GP, FM, EP, and IR collected data and revised the manuscript. CO reviewed the literature and revised the article. DM conceived the study and drafted and critically reviewed the paper. All authors approved the final manuscript as submitted and agree to be accountable for all aspects of the work.

## Conflict of Interest

The authors declare that the research was conducted in the absence of any commercial or financial relationships that could be construed as a potential conflict of interest.

## Publisher's Note

All claims expressed in this article are solely those of the authors and do not necessarily represent those of their affiliated organizations, or those of the publisher, the editors and the reviewers. Any product that may be evaluated in this article, or claim that may be made by its manufacturer, is not guaranteed or endorsed by the publisher.
