# Corrigendum: MIS-C Treatment: Is IVIG Always Necessary?

**DOI:** 10.3389/fped.2021.826518

**Published:** 2022-02-14

**Authors:** Francesco Licciardi, Letizia Baldini, Marta Dellepiane, Carlotta Covizzi, Roberta Mogni, Giulia Pruccoli, Cecilia Orsi, Ivana Rabbone, Emilia Parodi, Federica Mignone, Davide Montin

**Affiliations:** ^1^Department of Pediatrics and Public Health, Università degli Studi di Torino, Turin, Italy; ^2^Ospedale Infantile Regina Margherita, Città della Salute e della Scienza, Turin, Italy; ^3^Postgraduate School of Pediatrics, Università degli Studi di Torino, Turin, Italy; ^4^Department of Health Sciences, University of Piemonte Orientale, Novara, Italy

**Keywords:** MIS-C, SARS-CoV-2, therapy, IVIG (intravenous immunoglobulin) administration, steroid

In the original article, there was an error. Incorrect reference numbers and authors names were cited in the text. “2, 9, 12” has been updated to “2, 11, 13,” “…ICU stay (11); Del Borrello et al. and Sacco et al. found…” has been updated to “…ICU stay; Son et al. found…,” and “9, 12” has been updated to “2, 11.”

A correction has been made to **Discussion**, Paragraph Number 3:

“So far, three real-life retrospective studies have been conducted regarding the use of steroids to treat MIS-C (2, 11, 13). In two of them, the authors compared the outcomes of the patients treated with steroids and IVIG vs. IVIG alone. Ouldali et al. found that adding MP to IVIG led to a significant decrease of hemodynamic support needs and a reduction in length of ICU stay; Son et al. found that the patients treated with steroids and IVIG had a lower risk of new or persistent cardiovascular dysfunction compared to the ones treated with IVIG alone (2, 11).”

The authors apologize for this error and state that this does not change the scientific conclusions of the article in any way. The original article has been updated.

## Publisher's Note

All claims expressed in this article are solely those of the authors and do not necessarily represent those of their affiliated organizations, or those of the publisher, the editors and the reviewers. Any product that may be evaluated in this article, or claim that may be made by its manufacturer, is not guaranteed or endorsed by the publisher.

